# Targeting STAT3-VISTA axis to suppress tumor aggression and burden in acute myeloid leukemia

**DOI:** 10.1186/s13045-023-01410-y

**Published:** 2023-02-27

**Authors:** Jianshan Mo, Lin Deng, Keren Peng, Shumin Ouyang, Wen Ding, Linlin Lou, Ziyou Lin, Jianzheng Zhu, Jingwei Li, Qiyi Zhang, Pengyan Wang, Yuanzhen Wen, Xiaobing Chen, Peibin Yue, Jin-Jian Lu, Kai Zhu, Yongjiang Zheng, Yuanxiang Wang, Xiaolei Zhang

**Affiliations:** 1 National-Local Joint Engineering Laboratory of Druggability and New Drug Evaluation, Guangdong Key Laboratory of Chiral Molecule and Drug Discovery, School of Pharmaceutical Sciences, Sun Yat-Sen University, Guangzhou, 510006 China; 2 Innovation Practice Center, Changchun University of Chinese Medicine, Changchun, 130117 China; 3 Increasepharm (Hengqin) Innovative Medicine Institute Limited, Zhuhai, 519000 China; 4 Department of Medicine, Division of Hematology-Oncology, and Samuel Oschin Comprehensive Cancer Institute, Cedars-Sinai Medical Center, Los Angeles, CA 90048 USA; 5 State Key Laboratory of Quality Research in Chinese Medicine, Institute of Chinese Medical Sciences, University of Macau, Macao, 999078 China; 6 Department of Hematology, Institute of Hematology, The Third Affiliated Hospital of Sun Yat-Sen University, Guangzhou, 510630 China

**Keywords:** AML, VISTA, STAT3 inhibitor, Immunotherapy

## Abstract

The acute myeloid leukemia (AML) patients obtain limited benefits from current immune checkpoint blockades (ICBs), although immunotherapy have achieved encouraging success in numerous cancers. Here, we found that V-domain Ig suppressor of T cell activation (VISTA), a novel immune checkpoint, is highly expressed in primary AML cells and associated with poor prognosis of AML patients. Targeting VISTA by anti-VISTA mAb boosts T cell-mediated cytotoxicity to AML cells. Interestingly, high expression of VISTA is positively associated with hyperactive STAT3 in AML. Further evidence showed that STAT3 functions as a transcriptional regulator to modulate VISTA expression by directly binding to DNA response element of VISTA gene. We further develop a potent and selective STAT3 inhibitor W1046, which significantly suppresses AML proliferation and survival. W1046 remarkably enhances the efficacy of VISTA mAb by activating T cells via inhibition of STAT3 signaling and down-regulation of VISTA. Moreover, combination of W1046 and VISTA mAb achieves a significant anti-AML effect in vitro and in vivo. Overall, our findings confirm that VISTA is a potential target for AML therapy which transcriptionally regulated by STAT3 and provide a promising therapeutic strategy for immunotherapy of AML.

**Supplementary Information:**

The online version contains supplementary material available at 10.1186/s13045-023-01410-y.

## To the editor,

Despite encouraging responses elicited by immune checkpoint blockades (ICBs) have been shown in many types of cancers, the acute myeloid leukemia (AML) patients obtain little benefit from current ICBs [1, 2]. However, the mechanisms that determine immune evasion in AML are remain poorly understood. Herein, we found that V-domain immunoglobulin suppressor of T cell activation (VISTA) regulated by STAT3 is highly expressed in AML. VISTA is a novel immune checkpoint mediating immune evasion especially by impeding T cells activation [2–4]. Furthermore, we confirmed that combination of STAT3 inhibitor and VISTA mAb is a promising immunotherapeutic strategy for AML.

**Fig. 1:**
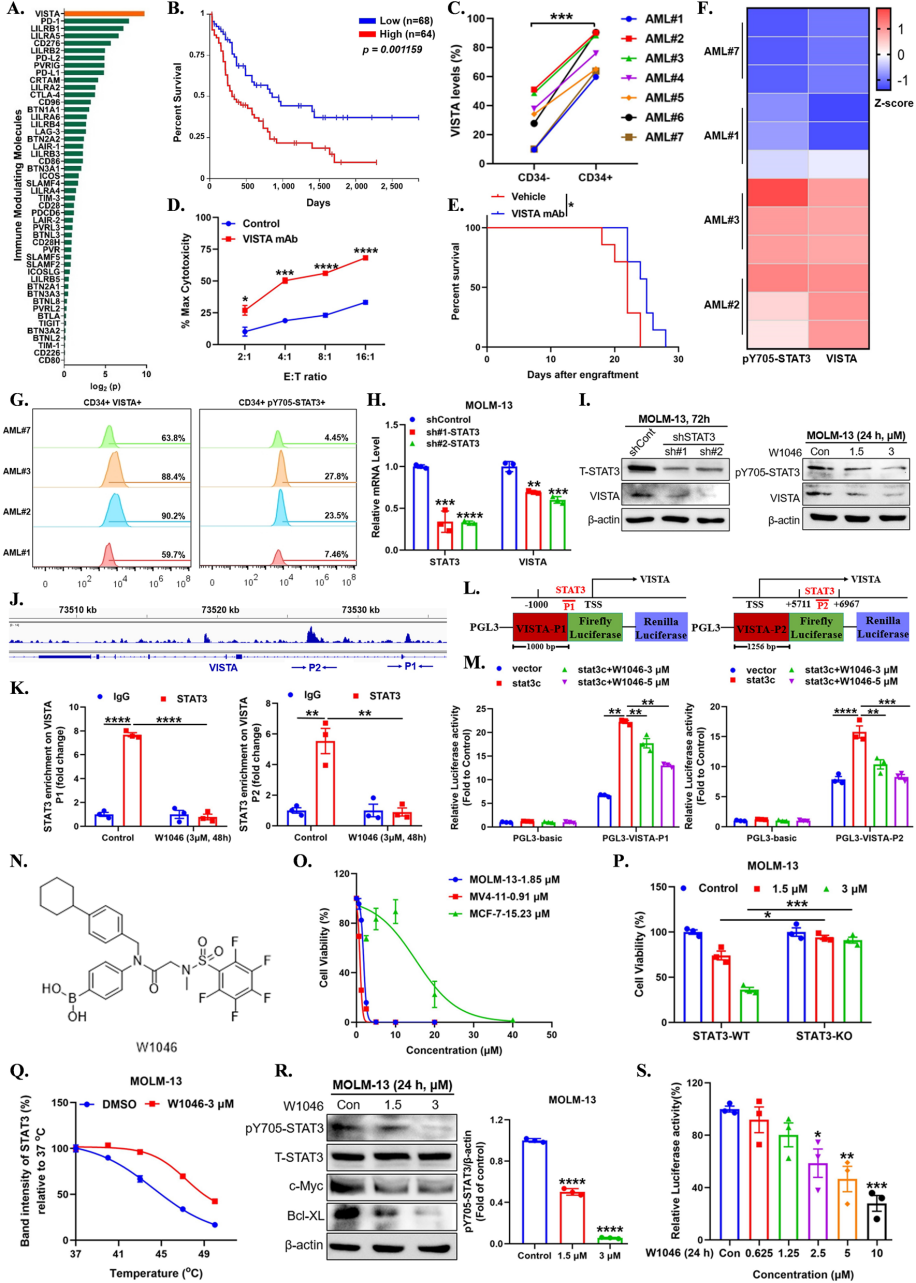
VISTA is highly expressed in AML cells and associated with poor prognosis of AML patients. **A** Correlation analysis between mRNA expression levels of immune-modulating molecules and the overall survival in AML patients (n = 132, divided into two groups based on gene expression) in GDC-TCGA database (https://xena.ucsc.edu) by Kaplan–Meier long-rank test. **B** Kaplan–Meier long-rank test of AML patients from GDC-TCGA database (n = 132, Low, n = 68; High, n = 64) with VISTA genes high or low expression levels. **C** VISTA expression changes in the CD34^+^ or CD34^−^ bone marrow-derived mononuclear cells from individual primary AML patient samples. **D** T cell-mediated cytotoxicity in co-culture system containing MOLM-13-EGFP/Luc cells and different amount of Jurkat cells in the presence of VISTA mAb (5 μg/mL). **E** Kaplan–Meier survival curve of intravenous C1498-EGFP/Luc mice (n = 7) treated with VISTA mAb. **F, G** VISTA levels and pY705-STAT3 levels in the CD34^+^ bone marrow-derived mononuclear cells of individual primary AML patient samples. **H** STAT3 were knockdown by specific shRNA for 48 h and the RT-PCR analysis of STAT3 and VISTA were measured in MOLM-13 cells. **I** STAT3 were knockdown by specific shRNA for 72 h or inhibited by W1046 for 24 h and the immunoblotting analysis of STAT3 and VISTA were measured in MOLM-13 cells. **J** Binding sites of STAT3 on VISTA gene were obtained from ChIP-seq data in GEO database (https://www.ncbi.nlm.nih.gov/gds/?term, GSM935276). **K** ChIP-qPCR analysis was performed to certify the binding between STAT3 and VISTA gene in MOLM-13 cells treated with DMSO or W1046 (3 μM) for 48 h. P1 represented the fragment from VISTA promoter, P2 represented the fragment from the first intron of VISTA gene. **L** The schematic of recombinant luciferase reporter construct containing 1000 bp of VISTA promoter or 1256 bp of the first intron of VISTA gene. **M** Relative luciferase activity of the P1 or P2 changed after STAT3 activation or treated with STAT3 inhibitor W1046 for 24 h in 293T cells. **N** Chemical structures of W1046. **O** Cell proliferation and IC_50_ values of MOLM-13 and Mv4-11with aberrantly active STAT3 and MCF-7 cells with low-active STAT3. **P** Cell viability of MOLM-13 cells with STAT3-WT or STAT3-KO treated with W1046 at different concentrations. **Q** melt curves of CETSA depicted degradation of STAT3 protein in MOLM-13 cells treated with W1046 or DMSO after being heated in the indicated temperature points. **R** Western blot was used to detect expression of pY705-STAT3, T-STAT3, c-Myc, and Bcl-XL after being treated with W1046 for 24 h in MOLM-13 cells. **S** The transcriptional activity measured by dual-luciferase reporter assay in 293T cells treated with W1046 at different concentration for 24 h. All data were presented as means ± SEM, n = 3. * P < 0.05, ** P < 0.01, *** P < 0.001

We found that VISTA was significantly higher in AML cells and was the most correlative immune checkpoint for overall prognosis of AML patients basing on TCGA database (Fig. 1A, B, Additional file 1: Fig. S1A), the same higher expression as in CD34 + AML cells and CD34- myeloid cells from bone marrow of AML patients (Fig. 1C, Additional file 1: Fig. S1B-S1C). Additionally, VISTA mAb significantly enhanced T cell-mediated cytotoxicity and prolonged survival of AML mice (Fig. 1D, E). These results together suggest that targeting VISTA might be a promising strategy for AML immunotherapy. As a member of B7 family [5], previous evidences suggesting that STAT3 activation is also correlated with expression of VISTA [6], but the molecular mechanisms remain a mystery. We found that high expression of VISTA was positive associated with STAT3 activation in AML cells (Additional file 1: Fig. S2A) and bone marrow of AML patients (Fig. 1F, G). Both genetic inhibition by specific shRNA and pharmacological inhibition by selective inhibitor W1046 of STAT3 significantly decreased the expression of VISTA at mRNA and protein level (Fig. 1H, I, Additional file 1: Fig. S2B-S2E). The same decrease of VISTA was found on AML cell membranes (Additional file 1: Fig. S2F-S2G). As a transcription factor closely associated with immune response, STAT3 regulates expression of immune genes transcriptionally [7, 8]. In this study, we found that STAT3 has two evident binding peaks in the promoter and the first intron of VISTA gene from Cistrome Data Browser database (Fig. 1J). ChIP-qPCR assay proved the bond between STAT3 and VISTA gene (Fig. 1K). Moreover, STAT3 significantly promoted transcription of VISTA, whereas the transcription activity was inhibited by loss-of-function mutation and selective STAT3 inhibitor W1046 or SH-4–54 (Fig. 1L, M, Additional file 1: Fig. S2H-S2J). The results suggest that STAT3 transcriptionally regulates VISTA by directly binding to promoter and intron region of VISTA.

**Fig. 2:**
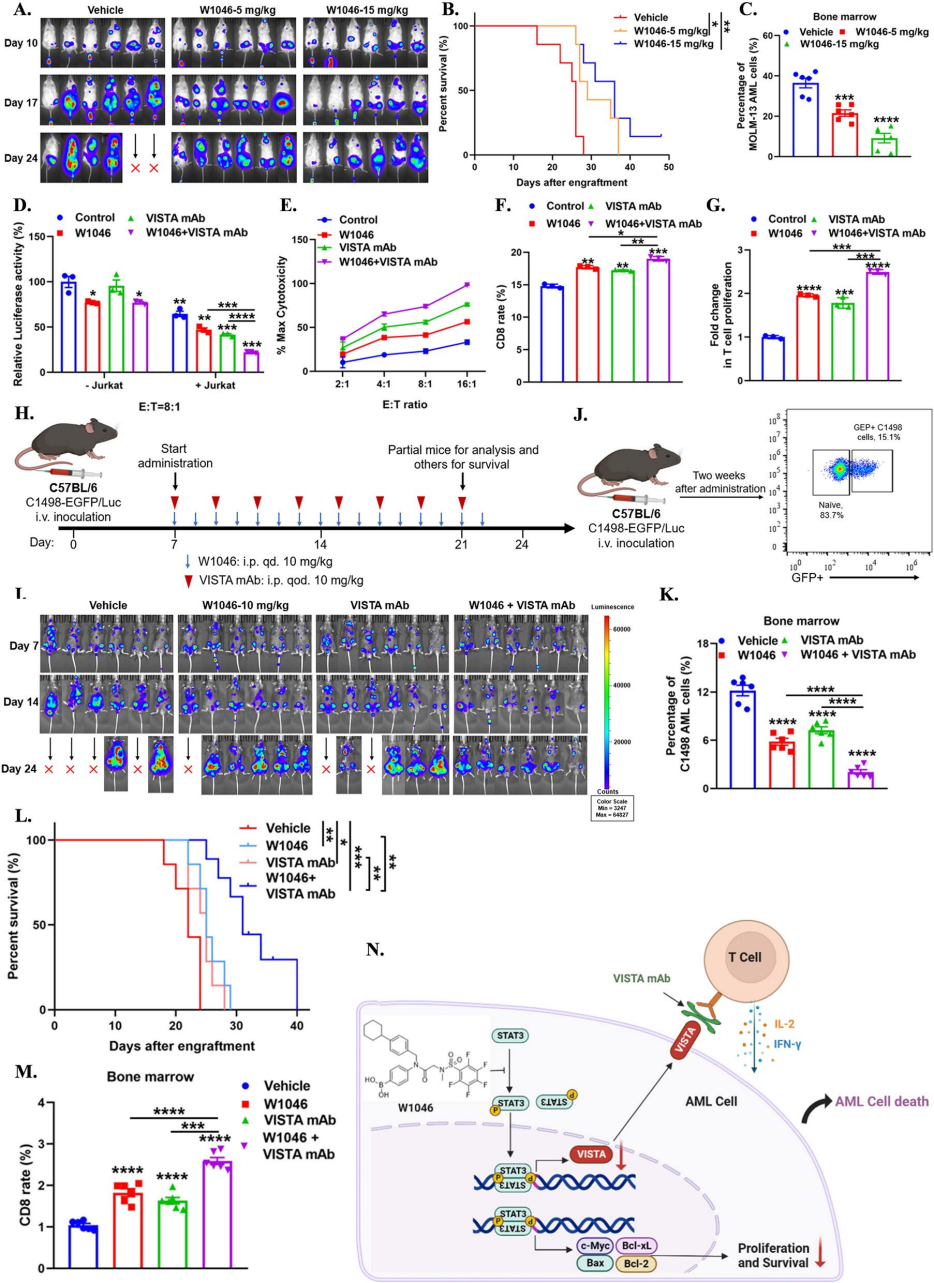
Combination of VISTA mAb and STAT3 inhibitor displayed potent anti-leukemia effect.** A** In vivo bioluminescence imaging of xenograft mouse models (n = 6) with MOLM-13-EGFP/Luc cells treated with W1046 at dosage of 5 mg/kg and 15 mg/kg. **B** Survival curve of xenograft mouse models (n = 7) with MOLM-13-EGFP/Luc cells treated with W1046 at dosage of 5 mg/kg and 15 mg/kg. **C** The residual GFP + MOLM-13 cells in bone marrow of mice (n = 6) were detected by flow cytometry after being treated with W1046 for two weeks. **D, E** T cell-mediated cytotoxicity in co-culture system containing MOLM-13-EGFP/Luc cells and effector cells at indicated E/T ratio. The pretreated or unpretreated MOLM-13-EGFP/Luc cells were co-cultured with or without activated Jurkat cells stimulated by anti-CD3 antibody (1 μg/mL) and anti-CD28 antibody (3 μg/mL) at indicated E/T ratio with or without VISTA mAb (5 μg/mL) for 24 h, then the survival cells were measured by Steady-Glo. **F** CD8^+^ T cells population changed in the co-cultured system. The activated PBMCs stimulated by anti-CD3 antibody (1 μg/mL) and anti-CD28 antibody (3 μg/mL) were co-cultured with MOLM-13 cells (with or without W1046 pretreatment) at a E/T ration of 20:1 with or without VISTA mAb for 72 h, then the CD8^+^ T cells population were detected by flow cytometry. E/T ratio, E: Effector cells (Jurkat cells or PBMCs); T, Target cells (AML cells). **G** CFSE dilution assay to measure the proliferation of T cells. The pretreated or unpretreated MOLM-13 cells were co-cultured with activated Jurkat cells stimulated by anti-CD3 antibody (1 μg/mL) and anti-CD28 antibody (3 μg/mL) and stained by CFSE, then combining with or without VISTA mAb for 72 h. **H** Illustration of procedure of therapy in AML mouse models. C57BL/6 mice were inoculated IV with 3 × 10^6^ C1498-EGFP/Luc cells and received indicated treatment. W1046 was intraperitoneally injected with 10 mg/kg every day and the VISTA mAb was intraperitoneally injected with 10 mg/kg once every other day. **I** In vivo bioluminescence imaging of xenograft mouse models (n = 6) with C1498-EGFP/Luc cells treated with W1046, VISTA mAb, or combination of W1046 and VISTA mAb. **J** Illustration of the procedure and identification of residual GFP + C1498 cells of mice (n = 6) by flow cytometry. **K** The residual GFP + C1498 cells in bone marrow of mice (n = 6) were detected by flow cytometry after being treated with W1046, VISTA mAb, or combination of W1046 and VISTA mAb for two weeks. **L** Survival curve of xenograft mouse models (n = 7) with C1498-EGFP/Luc cells treated with W1046, VISTA mAb, or combination of W1046 and VISTA mAb. **M** The infiltrating CD8^+^ T cells in bone marrow of mice (n = 6) were detected by flow cytometry after being treated with W1046, VISTA mAb, or combination of W1046 and VISTA mAb for two weeks. **N** Graphic Abstract (using the Biorender tools, an online platform for data analysis, https://biorender.com). Data were presented as mean ± SEM. *P < 0.05, **P < 0.01, ***P < 0.001

Basing on above results, we assumed STAT3 inhibitor can act as an immunomodulator in AML immunotherapy. On this account, we designed a novel STAT3 inhibitor W1046 (Fig. 1N), which we first introduced the pharmacophore of boronic acid targeting SH2 domain of STAT3 in the STAT3 inhibitor design [9]. W1046 significantly inhibited AML cells with hyperactive STAT3 but was lacked of sensitivity in MCF-7 cells with low STAT3 activation and in MOLM-13 cells with STAT3 deletion by CRISPR-Cas9 (Fig. 1O, P, Additional file 1: Fig. S3B). W1046 occupied the pivotal core of SH2 domain and elevated thermal stability of STAT3 protein (Fig. 1Q, Additional file 1: Fig. S3A, S3C). Further study showed W1046 significantly inhibited the phosphorylation and transcription of STAT3, but had no or slight effect on the phosphorylation of STAT1, STAT5, and upstream kinase JAK2 (Fig. 1R, S, Additional file 1: Fig. S3D-S3F). W1046 significantly inhibited proliferation and induced apoptosis both in AML cells lines and in primary AML patient samples (Additional file 1: Fig. S4A-S4F). Notably, W1046 remarkedly suppressed leukemia aggression, reduced leukemia burden and prolonged the survival of leukemia mice dosage-dependently (Fig. 2A–C, Additional file 1: Fig. S4G-S4H). Above results indicated that W1046 displays potent therapeutic efficacy on AML in vitro and in vivo as a novel STAT3 inhibitor.

Emerging evidences reported that combination of immunomodulator and immune checkpoint blockade generally has synergistic effect and maximizes benefits [10–12]. Given that the potential role of VISTA in AML immunotherapy, we next explored whether combination of STAT3 inhibitor W1046 with VISTA blockade would be a promising therapeutic strategy for AML. Firstly, we showed that combination of W1046 and VISTA mAb dramatically elevated T cell cytotoxicity in vitro (Fig. 2D, E, Additional file 1: Fig. S5A-S5B). Further study showed that combination of W1046 and VISTA mAb significantly upregulated secretion of IFN-γ and IL-2, promoted T cells proliferation, increased CD4^+^ T cells and CD8^+^ T cells (Fig. 2F, G, Additional file 1: Fig. S5C-S5J). Notably, combination of W1046 and VISTA mAb remarkedly suppressed leukemia aggression, reduced leukemia burden and prolonged the survival of leukemia mice by elevating infiltration of cytotoxic T cell in vivo (Fig. 2H–M, Additional file 1: Fig. S5K-S5L). Taken together, we testified that combination of W1046 and VISTA mAb displays significant anti-leukemia effect by decreasing VISTA and then enhancing T cells activation (Fig. 2N). The combined tactics would be a promising therapeutic strategy for AML.

Overall, our study firstly suggested that VISTA is a promising target for AML immunotherapy, regulated by STAT3 transcriptionally. A novel and selective STAT3 inhibitor W1046 was discovered and showed significant anti-tumor efficacy in AML. Combination of VISTA mAb and STAT3 inhibitor demonstrated remarkable anti-leukemia efficacy in AML by restoring T cells activation in vitro and in vivo, which shed a light on a new therapeutic strategy for AML therapy.

## Supplementary Information

**Additional file 1:** Supplementary figures S1–S5 and supplementary materials and methods.

## Data Availability

The datasets generated during and/or analyzed during the current study are available from the corresponding author on reasonable request.

## Abbreviations

AML: Acute myeloid leukemia
VISTA: V-domain Ig suppressor of T cell activation
ICBs: Immune checkpoint blockades
STAT3: Signal transducer and activator of transcription 3
