# Supplementary material for: Targeting STAT3-VISTA axis to suppress tumor aggression and burden in acute myeloid leukemia
Source: J Hematol Oncol. 2023 Feb 27;16:15. doi: 10.1186/s13045-023-01410-y (PMC9969711; doi:10.1186/s13045-023-01410-y)
Supplement: Supplementary file 1 — Additional file 1: Supplementary figures S1–S5 and supplementary materials and methods. [file 13045_2023_1410_MOESM1_ESM.docx]

**Supplementary Information for**

**Targeting STAT3-VISTA axis to suppress tumor aggression and burden in acute myeloid leukemia**

Jianshan Mo^1#^, Lin Deng^1#,^ Keren Peng^1#^, Shumin Ouyang^1^, Wen Ding^1^, Linlin Lou^1^, Ziyou Lin^1^, Jianzheng Zhu^1^, Jingwei Li^1^, Qiyi Zhang^1^, Pengyan Wang^2^, Yuanzhen Wen^3^, Xiaobing Chen^3^, Peibin Yue^4^, JinJian Lu^5^, Kai Zhu^2^, Yongjiang Zheng ^6*^, Yuanxiang Wang ^1*^, Xiaolei Zhang ^1^*

1. National-Local Joint Engineering Laboratory of Druggability and New Drug Evaluation, Guangdong Key Laboratory of Chiral Molecule and Drug Discovery, School of Pharmaceutical Sciences, Sun Yat-sen University, Guangzhou, 510006, China
2. Innovation Practice Center, Changchun University of Chinese Medicine, Changchun, 130117, China,
3. Increasepharm (Hengqin) Innovative Medicine Institute Limited, Zhuhai, 519000, China
4. Department of Medicine, Division of Hematology-Oncology, and Samuel Oschin Comprehensive Cancer Institute, Cedars-Sinai Medical Center, Los Angeles, CA, 90048, USA
5. Department of Pharmaceutical Sciences, Faculty of Health Sciences, University of Macau, Macao 519000, China,
6. Department of Hematology, Institute of Hematology, The Third Affiliated Hospital of Sun Yat-Sen University, Guangzhou, 510630, China

# These authors contributed equally.

*To whom correspondence should be addressed:

E-mail: zhangxlei5@mail.sysu.edu.cn (Xiaolei Zhang), Wangyx95@mail.sysu.edu.cn (Yuanxiang Wang), zhengyj5@mail.sysu.edu.cn (Yongjiang Zheng)

**Supplementary Materials and Methods**

**Cell lines and cell culture**

The human acute myeloid leukemia (AML) cell line Mv4-11, T lymphoblast cell line Jurkat, bladder cancer cell line 5637, mouse AML cell line C1498, and human embryonic kidney (HEK) cell line 293T were from American Type Culture Collection (ATCC, VA, USA), human AML cell line MOLM-13 was from AddexBio (San Diego, CA, USA). AML cell lines (MOLM-13 and Mv4-11), C1498, Jurkat and 5637 cell lines were cultured in RPMI-1640 (Gibco, Carlsbad, CA, USA) containing 10% fetal bovine serum (FBS, Gibco, Carlsbad, CA, USA) and 1% penicillin-streptomycin (P.S., Gibco, Carlsbad, CA, USA). 293T cell line was cultured in DMEM (Gibco, Carlsbad, CA, USA) containing 10% FBS and 1% PS. All cell lines were incubated in a humidified 5% CO_2_ incubator at 37 ^o^C.

**Primary human AML samples**

Primary human AML samples were obtained from the Third Affiliated Hospital of Sun Yat-Sen University (Guangzhou, China). Informed consent was obtained under protocols reviewed and approved by the Human Ethics Committee of the Third Affiliated Hospital of Sun Yat-Sen University (Guangzhou, China). All patient samples were purified using Ficoll density centrifugation and cultured in RPMI1640 media plus 20% FBS, ITS Solution (Gibco, Carlsbad, CA, USA), and 20% supernatant of the 5637 cells. Subsequently, the primary AML patient samples were used in activity evaluation, co-culture and flow cytometry analysis for protein differential expression.

**Cell viability and proliferation assay**

For the CCK8 assay, cells were seeded into 96-well plates with a density of 10000 cells per well. After 24 h, the cells were treated with W1046 and cultured in a cell incubator for 72 h， then Cell Counting Kit-8 (CCK-8, B34304, Bimake, Houston, TX, USA) was added into a 96-well plate and incubated for 1~4 h at 37 ^o^C. The cell viability was measured by a microplate reader (FLUOstar Omega-ACU, Ortenburg, Germany) for obtaining an OD value at 450 nm. The percentage of growth was calculated as Cell viability (%) = [A (Compound +) – A (Blank)] / [A (Compound -) – A (Blank)] × 100%. Each experiment was done thrice independently.

For EdU (5-Ethynyl-2’-deoxyuridine) assay, cells were seeded into 6-well plates with an appropriate density, after being treated with DMSO, W1046 for 3 days in indicated concentrations, the EdU assay was performed according to the manufacturer’s protocol of BeyoClick^TM^EdU-555 Cell Proliferation Kit (C0075S, Beyotime, Shanghai, China). Cells were treated with the corresponding concentration of EdU reagent for 2 h in a cell incubator at 37 ℃, 5% CO_2_, then discarded culture medium and added into 4% paraformaldehyde for fixation at room temperature for 15 min. After washing with PBS containing 3% BSA for 5 min twice, cells were permeated with 0.3% TritonX-100 for 15 min at room temperature. After permeating, the cells were washed twice and dyed with EdU reaction solution for indicating proliferation. The cell proliferation was detected by flow cytometry.

**Cell apoptosis assay**

Cell apoptosis induced by W1046 was detected by an Annexin V-FITC Apoptosis Detection Kit I (BestBio, Shanghai, China) according to the manufacturer’s instruction, and followed by cytometry analysis to detect the percentage of apoptotic cells. In addition, the protein levels relative to apoptosis were detected by western blot.

**Cellular thermal shift assay (CETSA)**

To determine the targeting of W1046 with STAT3 protein intracellularly, cells were cultured in a 15 cm culture dish and treated with W1046 or DMSO for 3 h when the cells with 70% to 80% confluence, then cells were harvested and washed twice with PBS, followingly suspended in 1 mL PBS (containing PMSF and phosphatase inhibitor cocktail) and subsequently divided into 0.2 mL PCR tubes with 100 μL per tube. Each tube was heated at the designated temperature point for 2 min, and the tubes were removed and cooled to room temperature immediately after heating. The tubes were frozen in liquid nitrogen immediately. To lyse cells, the tubes were performed with three freeze and thaw cycles in liquid nitrogen and at room temperature. The cell lysates were centrifuged at 20,000 × g for 20 min at 4 °C. Then the supernatants of cell lysates were heated at 100 ^o^C after adding into loading buffer, subsequently the samples were analyzed by western blot.

**Virus construction and infection**

For lentivirus packaging, plasmid constructs PLKO.1-sh-STAT3, lentiCRISPRv2-sgSTAT3, and plenti-EGFP-Luc were mixed with psPAX2 and PMD2.G (Addgene, Watertown, MA, USA) at a ratio of 4:3:1 and co-transfected into 293T cells using Hieff TransTM Liposomal Transfection Reagent (YEASEN, Shanghai, China). The supernatant containing packaging virus was collected 48 h post-transfection and used for infecting the AML cells, followed by screening using 1~3 μg/mL puromycin to construct stable transfected cells. The sequences of STAT3 knockdown were listed as supply table 1:

| Primer | Sequence (5’-3’) |
| --- | --- |
| sh#1-STAT3 Forward | CCGGGCACAATCTACGAAGAATCAACTCGAGTT  GATTCTTCGTAGATTGTGCTTTTTG |
| sh#1-STAT3 Reverse | AATTCAAAAAGCACAATCTACGAAGAATCAAC  TCGAGTTGATTCTTCGTAGATTGTGC |
| sh#2-STAT3 Forward | CCGGGCAAAGAATCACATGCCACTTCTCGAGAA  GTGGCATGTGATTCTTTGCTTTTTG |
| sh#2-STAT3 Reverse | AATTCAAAAAGCAAAGAATCACATGCCACTTCT  CGAGAAGTGGCATGTGATTCTTTGC |

**CRISPR/Cas9-mediated gene knockout in AML cells**

For abrogating STAT3 in MOLM-13 completely, the lentiCRISPRv2 containing sgRNA-expressing and Cas9-expressing was used. After an optimized guide RNA (gRNA) targeting the exon2 of STAT3 designed by an online CRISPR design tool (<http://crispor.tefor.net/>) was cloned into lentiCRISPRv2 vector and lentivirus packaging, the virus was applied to infect MOLM-13 cells. 48 h later, 2 μg/mL puromycin was used for selecting for 2 weeks. Then the puromycin-resistant cells were diluted into a 96-well plate with a density of a single cell per well. The cell clones were expanded and confirmed by western blot analysis. The gRNA used to knockout STAT3 were listed as table 2:

| Primer | Sequence (5’-3’) |
| --- | --- |
| Forward primer | CACCGCAGCTTGACACACGGTACC |
| Reverse primer | AAACGGTACCGTGTGTCAAGCTGC |

**Luciferase reporter assays and mutagenesis assay**

From the ChIP-seq database, we obtained a binding site of STAT3 located in the first intron of the VISTA gene (<http://cistrome.org/db/#/>, GSM935276). A 1256 bp fragment of the VISTA gene (nucleotides +5711 bp to +6967 bp relative to the transcription initiation site) was amplified by PCR from 293T cell line genomic DNA and inserted into PGL3-basic vector (Promega, Madison, Wisconsin, USA). To get the specific motif STAT3 binding to, we interrogated the sequence into the JASPAR database (<https://jaspar.genereg.net/>) and selected a putative binding motif, and followed by mutagenesis in designated nucleotide using Fast Mutagenesis System Kit (FM111-01, TRANS, Beijing, China) according to the manufacturer’s instruction. Luciferase activity was measured by Steady-glo kit (Promega, Madison, Wisconsin, USA), and the β-gal activity was used for normalizing the firefly luciferase activity and reflected the transcriptional level. All experiments were performed in triplicates independently. The primers for constructing mutant VISTA reporter gene plasmid were described in table 3.

| Primer | Sequence (5’-3’) |
| --- | --- |
| Forward primer | TTGTCCTCACTGGCCACACCGGCCCCAGGGC |
| Reverse primer | GGTGTGGCCAGTGAGGACAAAGGGCATCTCA |

**ChIP-qPCR**

MOLM-13 cells were treated by vehicle or W1046 (3 μM) for 48 h before being harvested. The anti-STAT3 primary antibody (10253-2-AP, Proteintech, Wuhan, Hubei, China) was used to immunoprecipitate protein complexes containing STAT3. Putative Stat3-binding-site sequences in the first intron of VISTA were detected from the immunoprecipitates using RT-PCR with the primers described as table 4.

| Primer | Sequence (5’-3’) |
| --- | --- |
| Forward primer | CTGAGATGCCCTTTGTCCTCAC |
| Reverse primer | ATCCCCCAGGATGGTCCAC |

**Western blot and immunoprecipitation**

Cells were lysed with RIPA lysis buffer (Beyotime, Shanghai, China) supplemented with protease inhibitors (Beyotime, Shanghai, China) and phosphatase inhibitors cocktail (Bimake, Houston, TX, USA) after being treated with the indicated concentrations of the drug. Whole cell lysates were measured using a BCA Protein Assay Kit (Thermo Fisher, Waltham, MA, USA) and denatured for 5 min by heating in 99 ^o^C. The protein samples were electrophoresed through a 5%~12% SDS-PAGE gel and transferred to polyvinylidene fluoride (PVDF) membranes (Merck Millipore, Darmstadt, Germany), and the membranes were blocked and probed by indicated primary antibodies and secondary HPR-conjugated antibodies. After that, the membranes were imaged by a chemiluminescence instrument (Bio-Rad, Hercules, CA, USA).

**RNA extraction and quantitative real-time PCR**

Total cellular RNA was extracted by using Trizol (YEASEN, Shanghai, China) according to the manufacturer’s protocol and cDNAs were obtained from 2 μg total RNA reverse-transcription by using Hifair® Ⅱ 1st Strand cDNA Synthesis Kit (YEASEN, Shanghai, China) according to the manufacturer’s protocol. Quantitative real-time PCR (QPCR) analyses were conducted by a CFX Connect^TM^ real-time system (Bio-Rad, Hercules, CA, USA) with SYBR Green master mix (YEASEN, Shanghai, China). The specific primers were used to detect mRNA expression levels of STAT3 and VISTA, and the β-actin was used to as normalization. The specific primers sequences were listed as table 5:

| Primer | Sequence (5’-3’) |
| --- | --- |
| STAT3 Forward | CTGCCCCATACCTGAAGACC |
| STAT3 Reverse | TCCTCACATGGGGGAGGTAG |
| VISTA Forward | CACCAGAAGTTCCTCTGCGCGT |
| VISTA Reverse | CGTCTTGTAGAAGGTCACATCGTGC |
| β-actin Forward | ACTCTTCCAGCCTTCCTTCC |
| β-actin Reverse | CGTACAGGTCTTTGCGGATG |

**Flow cytometry analysis**

Primary antibodies including anti-human CD45-Brilliant Violet 421^TM^ (Biolegend, San Diego, CA, USA), anti-human CD34-Percp (Biolegend, San Diego, CA, USA), anti-human CD3-FITC (Biolegend, San Diego, CA, USA), anti-human CD4-Alexa Fluor® 700 (Biolegend, San Diego, CA, USA), anti-human CD8-APC (Biolegend, San Diego, CA, USA), anti-human VISTA-PE/Cyanine 7 (Invitrogen, Carlsbad, CA, USA), anti-mouse CD45-FITC (Biolegend, San Diego, CA, USA), anti-mouse CD25-PE/Cyanine 7 (Biolegend, San Diego, CA, USA), anti-mouse CD3-APC/Cyanine 7 (Biolegend, San Diego, CA, USA), anti-mouse CD4- Alexa Fluor® 700 (Biolegend, San Diego, CA, USA), CD4-PE (Biolegend, San Diego, CA, USA), anti-mouse CD8-APC (Biolegend, San Diego, CA, USA), anti-mouse VISTA-APC (Biolegend, San Diego, CA, USA), anti-phosphorylated STAT3 (Try705)-PE (Biolegend, San Diego, CA, USA) were used. The cells were stained by corresponding antibodies and detected by flow cytometry. The FlowJo software was used for the analysis of flow cytometry data.

**T cell co-culture and cytotoxic assay**

For cytotoxicity assay, the MOLM-13-EGFP/Luc cells were pretreated with W1046 (3 μM) for 24 h. The Jurkat cells were plated into a 96-well plate at different densities as an indicated ratio with leukemia cells (effected cells to target cells, E: T) and stimulated with 2.5 μg/mL anti-CD3 (Biolegend, San Diego, CA, USA) and 3 μg/mL anti-CD8 for 24 h, then, 2×10^4^ cells treated or untreated MOLM-13-EGFP/Luc cells and anti-VISTA antibodies or control IgG were added and co-culture for another 24 h. The Steady-Glo were added to measure the activity of luciferase to indicate the viability of alive leukemia cells. In addition, another group that has only MOLM-13-EGFP/Luc cells with or without W1046 or anti-VISTA antibody treatment were evaluated for the direct antitumor activity and as normalization.

For T cells activation assay, the pretreated or unpretreated MOLM-13 cells were co-cultured with human PBMCs which had been stimulated by anti-CD3 and anti-CD28 antibodies at a E:T ratio of 4:1 for 72 h. Subsequently, the T cells were stained with anti-CD3, anti-CD4 and anti-CD8 antibodies and analyzed by flow cytometry to represent the activation of T cells.

For the mouse, leukemia/T cells co-culture system, spleen cells from wild-type C57BL/6 mice were activated by anti-CD3 and anti-CD8 antibodies for 24 h and subsequently plated into a 96-well plate, and then added into VISTA mAb or IgG control and co-cultured with C1498 or C1498-EGFP/Luc cells which had been treated or untreated with W1046 at the indicated E:T ratio for 24 h or 72 h. The relative firefly luciferase activity was detected and T cell subgroups were analyzed by flow cytometry to indicate the T cells’ cytotoxicity.

**T-cell proliferation**

To assess the T-cell proliferation, the Jurkat cells were activated with anti-CD3 and anti-CD28 antibodies for 24 h. Then the activated Jurkat cells were stained with CFSE and co-cultured with AML cells. After co-culturing 72 h, the proliferative Jurkat cells were analyzed by flow cytometry.

***In vivo* Studies**

The animal procedures were approved by the Institutional Animal Care and Use Committee (IACUC), Sun Yat-Sen University (SYSU-IACUC-2022-000185) and conducted following the Guide for the Care and Use of Laboratory Animals. For the MOLM-13-EGFP/Luc xenograft model, 5×10^6^ MOLM-13-EGFP/Luc cells were resuspended in 100 µl PBS and subsequently intravenously injected into the tail vein of NOD-SCID mice (6~8 weeks). The mice were allocated to each group and treated intraperitoneally (i.p.) with the vehicle, 5 mg/kg W1046 or 15 mg/kg W1046 for once a day. The *in vivo* bioluminescence imaging was used to measure the progress of AML in mice and partial mice for survival analysis.

For the immunocompetent AML mouse model, 3×10^6^ C1498-EGFP/Luc cells were resuspended in 100 µl PBS and subsequently intravenously injected into the C57BL/6 mice (6~8 weeks). The mice were divided into four groups and treated with vehicle, W1046, anti-VISTA antibody or combination of W1046 and anti-VISTA antibody. W1046 was intraperitoneally (i.p.) injected with 10 mg/kg for once a day, and the anti-VISTA antibody was intraperitoneally (i.p.) injected with 10 mg/kg once every two days. The *in vivo* bioluminescence imaging was used to measure the progress of AML mice at different time points. After being treated for two weeks, partial mice were euthanized and analyzed for residual AML cells and T cell activation, and the other mice were sequentially treated for survival analysis.

**Statistical analysis**

Statistical analysis was performed on mean values using Prism (GraphPad Software, USA). The significance of differences between groups was determined via the unpaired t-test as *P<0.05, **P<0.01, ***P<0.001.

**Supplementary Figures**





**Supplementary Figure S1. | VISTA highly expression in AML cells. (A)** The expression of VISTA mRNA levels in patients with AML or healthy donors in TCGA database (http://gepia.cancer-pku.cn/index.html). patients with AML, n = 173; normal, n = 70. **(B)** VISTA levels detected by flow cytometry on the bone marrow-derived mononuclear cells from AML patients. **(C)** VISTA levels in the CD34+ or CD34- bone marrow-derived mononuclear cells from AML patients. Data were presented as mean ± SEM. *P < 0.05, **P < 0.01, ***P < 0.001.





**Supplementary Figure S2. | STAT3 regulates expression of VISTA transcriptionally. (A)** Correlation analysis of gene expression between VISTA and STAT3 in patients with AML from a GEO database (https://www.ncbi.nlm.nih.gov/gds/). **(B)** STAT3 were knockdown by specific shRNA for 48 h and the RT-PCR analysis of STAT3 and VISTA were measured in Mv4-11 cells. **(C)** MOML-13 and Mv4-11 cells were treated with W1046 at different concentrations and the mRNA levels of VISTA were measured by QPCR. **(D-E)** STAT3 were knockdown by specific shRNA for 72 h or inhibited by W1046 for 24 h and the immunoblotting analysis of STAT3 and VISTA were measured in Mv4-11 and C1498 cells. **(F-G)** The protein levels of VISTA on cell membrane were detected by flow cytometry after STAT3 knockdown by specific shRNA for 72 h or being inhibited by STAT3 inhibitor W1046 for 24 h in MOLM-13, Mv4-11 and C1498 cells. **(H)** Relative luciferase activity of the P1 or P2 changed after STAT3 activation or treated with another STAT3 inhibitor SH-4-54 for 24 h in 293T cells. **(I)** The potential binding motif on the first intron of VISTA gene STAT3 binding to was predicted by JASPAR (https://jaspar.genereg.net/). The mutant bases of motif were shown in red fonts. **(J)** Relative luciferase activity of the P2-WT or P2-Mut changed after STAT3 activation in 293T cells. Data were presented as mean ± SEM, n=3. *P < 0.05, **P < 0.01, ***P < 0.001.





**Supplementary Figure S3. | W1046 is a novel and potent inhibitor for STAT3. (A)** The computational modeling of STAT3 binding to STAT3 protein. **(B)** The knockout efficiency was measured by western blot after STAT3 deletion mediated by CRISPR-Cas9 in MOLM-13 cells. **(C)** CETSA depicted degradation of STAT3 protein in MOLM-13 cells treated with W1046 or DMSO after being heated in the indicated temperature points. **(D)** Mv4-11 and C1498 cells were treated with W1046 at different concentrations for 24 h, and the western blot was used to detect expression of pY705-STAT3, T-STAT3, c-Myc, and Bcl-XL. **(E)** The immunoblotting the western blot analysis of pY701-STAT1, STAT1, pY694-STAT5 and STAT5 in MOLM-13 and Mv4-11 cells after being treated with W1046 at different concentrations for 24 h. **(F)** The immunoblotting the western blot analysis of p-JAK2 and JAK2 in MOLM-13 and Mv4-11 cells after being treated with W1046 at different concentrations for 24 h. Data were presented as mean ± SEM, n=3. *P < 0.05, **P < 0.01, ***P < 0.001.





**Supplementary Figure S4. | W1046 showed significant anti-leukemia efficacy in AML. (A)** Cell proliferation and IC_50_ values of C1498 and H9C2 cell lines and AML cells derived from bone marrow of primary AML patients. **(B-C)** Proliferation of MOLM-13 and Mv4-11 cells treated with W1046 at different concentrations for 72 h were measured by EdU assay. **(D-E)** MOLM-13, Mv4-11 cells and AML cells derived from bone marrow of primary AML patients were treated with W1046 at different concentrations for 72 h, and the apoptotic cells were measured by PI and Annexin V-FITC. **(F)** MOLM-13 and Mv4-11 cells were treated with W1046 at different concentration for 72 h, and the expression of relevant apoptotic protein cleavaged-Caspase-7, Bax and Bcl-2 were detected by western blot. **(G)** Blood smears were stained with Giemsa stain and representative images were shown. The arrows indicated immature cells and the scale bars equal 400 µm. Histopathology of spleen were analyzed by HE staining and the scale bars equal 200 µm. **(H)** The residual GFP+ MOLM-13 cells in spleen of mice (n = 6) were detected by flow cytometry after being treated with W1046 for two weeks. Data were presented as mean ± SEM. *P < 0.05, **P < 0.01, ***P < 0.001.





**Supplementary Figure S5. | Combination of VISTA mAb and STAT3 inhibitor enhanced T cell activation. (A-B)** T cell-mediated cytotoxicity in co-culture system containing C1498-EGFP/Luc cells and effector cells at indicated E/T ratio. The pretreated or unpretreated C1498-EGFP/Luc cells were co-cultured with or without activated splenocytes stimulated by anti-CD3 antibody (1 μg/mL) and anti-CD28 antibody (3 μg/mL) at indicated E/T ratio with or without VISTA mAb (5 μg/mL) for 24 h, then the survival cells were measured by Steady-Glo. **(C-D)** The IFN-γ or IL-2 secretion were measured by ELISA. MOLM-13 (with or without W1046 pretreatment) were co-cultured with PBMCs stimulated by anti-CD3 antibody (1 μg/mL) and anti-CD28 antibody (3 μg/mL) with or without VISTA mAb for 72 h. **(E-F)** The VISTA levels on MOLM-13 cell membrane in the co-cultured system were detected by flow cytometry after indicated treatment. **(G-I)** CD4+ and CD8+ T cells population changed in the co-cultured system. The activated PBMCs stimulated by anti-CD3 antibody (1 μg/mL) and anti-CD28 antibody (3 μg/mL) were co-cultured with MOLM-13 cells (with or without W1046 pretreatment) at a E/T ration of 20:1 with or without VISTA mAb for 72 h, then the CD4+ and CD8+ T cells population were detected by flow cytometry. **(J)** CFSE dilution assay to measure the proliferation of T cells. The pretreated or unpretreated MOLM-13 cells were co-cultured with activated Jurkat cells stimulated by anti-CD3 antibody (1 μg/mL) and anti-CD28 antibody (3 μg/mL) and stained by CFSE, then combining with or without VISTA mAb for 72 h. **(K)** IHC analysis showed the infiltrating CD8+ T cells in spleen of mice (n = 6) after being treated with W1046, VISTA mAb, or combination of W1046 and VISTA mAb for two weeks. **(L)** Histopathology analysis of bone marrow and spleen by HE staining. The scale bars equal 200 µm. E/T ratio, E: Effector cells (Jurkat cells or PBMCs); T, Target cells (AML cells). Data were presented as mean ± SEM. *P < 0.05, **P < 0.01, ***P < 0.001.
